# Lonicerae Japonicae Flos and Lonicerae Flos: A Systematic Pharmacology Review

**DOI:** 10.1155/2015/905063

**Published:** 2015-07-16

**Authors:** Yujie Li, Weiyan Cai, Xiaogang Weng, Qi Li, Yajie Wang, Ying Chen, Wei Zhang, Qing Yang, Yan Guo, Xiaoxin Zhu, Hainan Wang

**Affiliations:** ^1^Institute of Chinese Materia Medica, China Academy of Chinese Medical Sciences, No.16, Dongzhimen Nei Nanxiao Road, Dongcheng District, Beijing 100700, China; ^2^Department of Drug and Cosmetics Registration, China Food and Drug Administration, Xuanwumen Xidajie, Beijing 100053, China

## Abstract

Lonicerae japonicae flos, a widely used traditional Chinese medicine (TCM), has been used for several thousand years in China. *Chinese Pharmacopeia* once included Lonicerae japonicae flos of Caprifoliaceae family and plants of the same species named Lonicerae flos in general in the same group. *Chinese Pharmacopeia* (2005 Edition) lists Lonicerae japonicae flos and Lonicerae flos under different categories, although they have the similar history of efficacy. In this study, we research ancient books of TCM, 4 main databases of Chinese academic journals, and MEDLINE/PubMed to verify the origins and effects of Lonicerae japonicae flos and Lonicerae flos in traditional medicine and systematically summarized the research data in light of modern pharmacology and toxicology. Our results show that Lonicerae japonicae flos and Lonicerae flos are similar pharmacologically, but they also differ significantly in certain aspects. A comprehensive systematic review and a standard comparative pharmacological study of Lonicerae japonicae flos and Lonicerae flos as well as other species of Lonicerae flos support their clinical safety and application. Our study provides evidence supporting separate listing of Lonicerae japonicae flos and Lonicerae flos in *Chinese Pharmacopeia* as well as references for revision of relevant pharmacopeial records dealing with traditional efficacy of Lonicerae japonicae flos and Lonicerae flos.

## 1. Introduction

Lonicerae japonicae flos (also Jinyinhua in Chinese), a plant species in traditional Chinese medicine (TCM), has been widely used as a drug for several millennia with confirmed curative effects. It has been recorded in the* Chinese Pharmacopeia* (1963 Edition), limiting the therapeutic use of Lonicerae japonicae flos to the dried flower buds of* Lonicera japonica* Thunb., which belongs to Caprifoliaceae. In the 1977, 1985, 1990, 1995, and 2000 Editions of* Chinese Pharmacopeia*, three other plant sources were also listed in the category of Lonicerae japonicae flos including the dried flower buds or initial flowers of* Lonicera hypoglauca* Miq.,* Lonicera confusa* DC., and* Lonicera dasystyla* Rehd.* Chinese Pharmacopeia* (2005 Edition) lists Lonicerae japonicae flos and Lonicerae flos as independent items based on differences in medicinal history, plant morphology, medicinal properties, and chemical constituents, and the only plant source of Lonicerae japonicae flos is again limited to* Lonicera japonica* Thunb. Lonicerae flos has three sources of germplasm including* Lonicera macranthoides* Hand.-Mazz.,* Lonicera hypoglauca* Miq., and* Lonicera confusa* DC.* Chinese Pharmacopeia* (2010 Edition) adds* Lonicera fulvotomentosa* Hsu et S. C. Cheng to Lonicerae flos following the 2005 Edition, making the aforementioned 4 plant species legal for Lonicerae flos. However, the descriptions of flavor, meridian tropism (MT), functions, and indications are not different between Lonicerae japonicae flos and Lonicerae flos in* Chinese Pharmacopeia* although they have different sources.

In order to comprehensively review the pharmacological studies, we investigate the efficacy of Lonicerae japonicae flos and Lonicerae flos in ancient books of TCM and searched for the literatures both at home and abroad from the China Academic Journal Network Publishing Database of the China National Knowledge Infrastructure, Wanfang Database, China Biomedical Database, and MEDLINE/PubMed. A total of 2864 papers relevant to Lonicerae japonicae flos and Lonicerae flos are retrieved before August 2014, of which 514 dealt with pharmacological effects of main ingredients of Lonicerae japonicae flos and Lonicerae flos. The literatures are further collated to summarize the studies with clear information of the origin of species. Pharmacological advances are also systematically reviewed to provide references for scientific appreciation of Lonicerae japonicae flos and Lonicerae flos.

## 2. Traditional Records of Lonicerae Japonicae Flos [1]

Lonicerae japonicae flos is termed “Rendong” in ancient books of TCM. The* Collective Notes to Canon of Materia Medica* (around 480–498 AD) writes: “It grows everywhere and is classified into liane. It does not fade over winter and thus be named as Rendong.” Since then only the name “Rendong” has been recorded in all the medical books until Tang Dynasty. Subsequently in Song Dynasty,* The Prescriptions of Su and Shen* (960–1127 AD) and* Lvchanyan Materia Medica* (1220 AD) used the term “Lonicerae japonicae flos” as a herb also named as Rendong.

Before Ming Dynasty, the* Collective Notes to Canon of Materia Medica*,* Annotation of Materia Medica*, and* The Prescriptions of Su and Shen* only provided a brief description of Rendong and the recorded characteristics of the original plant including liane, opposite leaf, and leaf shape consistent with those of Caprifoliaceae. In Ming Dynasty, the* Compendium of Materia Medica* (1578 AD) offered the most detailed description of* Lonicera* flower that “it blossoms in March and April with the length of flower over 3 decimeters. One pedicel contains 2 flowers and each flower has 2 petals either large or narrow with half-edge structure. It has stamens and pistils. The early flower has white stamens, pistils, and petals that turn yellow in 2 to 3 days.” In the ancient Materia Medica,* An Illustrated Book of Plants* authored by Wu Qijun in Qing Dynasty included the ink drawings of Lonicerae japonicae flos with accurate proportion and precise morphology. In addition, the* Collected Essentials of Species of Materia Medica* written in Ming Dynasty also contains representative colored illustrations of Lonicerae japonicae flos, and the archives show clearly the typical morphological characteristics of Caprifoliaceae including liane, 1 pedicel containing 2 flowers, lip-like and white flowers, opposite leaf, ovate shape, and lobate and ovate shape.

In summary, the original plant morphology of Lonicerae japonicae flos is basically similar in the studies before Qing Dynasty. No records of the medicinal use of Lonicerae flos have been found in ancient books. Based on comparative plant morphology, only* Lonicera japonica* Thunb., among 21 varieties of* Lonicera*, is consistent with the morphological characteristics of traditional medicinal Lonicerae japonicae flos while the other 4 species in* Chinese Pharmacopeia* (2010 Edition) were markedly different in origin.

According to* Chinese Pharmacopeia* (2005 and 2010 Editions), both Lonicerae japonicae flos and Lonicerae flos are sweet in flavor and cold in nature, attributed to lung, heart, and stomach meridians. They clear heat, toxins, and certain external ailments. They are indicated for carbuncles and pyocutaneous disease, pharyngitis, erysipelas, heat toxins, blood dysentery, exogenous hot ailments, and febrile diseases.

## 3. Modern Pharmacological Studies on Lonicerae Japonicae Flos and Lonicerae Flos

Lonicerae japonicae flos grows mainly in Shandong, Shaanxi, Henan, and Hebei Provinces in China, among which the Pingyi County, Linyi City, Shandong Province, is the main area with the largest production. Lonicerae flos is mainly grown in the provinces located at the southern regions of Yangtze River such as Hunan Province, Sichuan Province, Guangdong Province, Guizhou Province, and Guangxi Province. Recent studies have been focused on the pharmacological effects of Lonicerae japonicae flos. In contrast, the studies of Lonicerae flos are comparatively limited and most studies focus on* Lonicera macranthoides* Hand.-Mazz., which exhibits basically similar pharmacological effects as those of Lonicerae japonicae flos.

### 3.1. Antibacterial Effects

#### 3.1.1. Lonicerae Japonicae Flos

Many pharmacologic studies have clearly confirmed the bacteriostatic and antibacterial effects of Lonicerae japonicae flos both* in vivo* and* in vitro*. Compared with other commonly seen antibacterial drugs, Lonicerae japonicae flos exhibits a broader antimicrobial spectrum, more powerful antibacterial activity, and inhibition of drug-resistant bacteria. The antibacterial activities of Lonicerae japonicae flos were detailed in Table 1.

Components of Lonicerae japonicae flos including water extract, alcohol extract, polysaccharide, and volatile oil can extensively inhibit Gram-negative bacteria and Gram-positive bacteria including* Streptococcus haemolyticus*,* Staphylococcus aureus*,* Salmonella* Typhi,* Klebsiella pneumoniae*,* Salmonella paratyphi*,* Vibrio cholerae*, oral pathogens,* Diplococcus intracellularis*,* Streptococcus pneumoniae*,* Mycobacterium tuberculosis*, and* Pseudomonas aeruginosa*. In particular, the water extracts of Lonicerae japonicae flos strongly inhibit* Escherichia coli* and* Staphylococcus aureus* but are comparatively weak against* Pseudomonas aeruginosa* and* Shigella flexneri* [2–12]. Based on* in vivo* antimicrobial tests, Lonicerae japonicae flos is fairly inhibitory to* Staphylococcus aureus* and* Diplococcus pneumoniae* but minimally active against other pathogens [13, 14]. A study reports that 10% Lonicerae japonicae flos extract has antibacterial effect against* Escherichia coli* and* Staphylococcus aureus*, which is equivalent to that of penicillin 100 *μ*mol/mL [15], indicating characteristic high antibacterial activity similar to that of antibiotics.

The components of Lonicerae japonicae flos including water-soluble polysaccharides have remarkable inhibitory activities against fungi such as dermatophytes,* Saccharomyces cerevisiae*,* Penicillium citrinum*,* Aspergillus niger*,* Cryptococcus neoformans*,* Fusarium moniliforme*,* Candida albicans*, and* Aspergillus* sp. In addition, the volatile oil of Lonicerae japonicae flos has proven to have antifungal activities [16–18].

Current clinical antimicrobial treatments are associated with a high frequency of multidrug resistance and widespread plasmid resistance. Studies suggest that Lonicerae japonicae flos extracts not only potently inhibit common pathogens but also significantly suppress drug-resistant bacteria. In addition, the water decoction effectively inhibits drug-resistant R plasmid of* Pseudomonas aeruginosa* and sensitizes* Pseudomonas aeruginosa* to single antibiotic. Mice administered with extracts of Lonicerae japonicae flos show improved multiantibiotic sensitivities [19–21]. Lonicerae japonicae flos kills several clinically common drug-resistant bacteria such as methicillin-resistant* Staphylococcus aureus* (MRSA), methicillin-resistant* Staphylococcus haemolyticus* (MRSH), methicillin-resistant* Staphylococcus epidermidis* (MRSE), and high-level aminoglycoside resistant (HLAR) bacteria to varying degrees, and the antibiotic effects are positively correlated with drug concentration [22]. These experimental results further enrich our understanding of the antibacterial activity of Lonicerae japonicae flos and provide scientific data of therapeutic efficacy against drug-resistant pathogens.

#### 3.1.2. Lonicerae Flos

Reports of efficacy and pharmacology of Lonicerae flos suggest several effective antifungal and antibacterial extracts (see Table 1).

The ethanol extracts, water extracting-alcohol precipitating solution, and water extracts of Lonicerae flos are strongly inhibitory against multiple pathogens such as* Pseudomonas aeruginosa*,* Bacillus subtilis*,* Staphylococcus aureus*,* Candida albicans*,* Escherichia coli*,* Salmonella* Typhi,* Shigella dysenteriae*, and* Pseudomonas aeruginosa* [23–25] and effectively protected* Staphylococcus aureus*-infected mice [26].

Lonicerae flos shows various antibacterial activities. The water extracts of* Lonicera macranthoides* Hand.-Mazz. are the most effective against* Staphylococcus aureus* and group B* Streptococcus* and effective against* Salmonella* Typhi,* Escherichia coli*, and* Shigella dysenteriae* but poorly effective against* Proteus vulgaris* [27]. Lonicerae flos (*Lonicera macranthoides* Hand.-Mazz.,* Lonicera hypoglauca* Miq.) from Sichuan has inhibitory effects against* Staphylococcus aureus*,* Streptococcus haemolyticus*,* Escherichia coli*,* Shigella flexneri*,* Salmonella* Typhimurium, and* Streptococcus pneumoniae*, but not against* Streptococcus haemolyticus* and* Salmonella* Typhimurium [28]. Although reports of antibacterial activity of Lonicerae flos vary, they still clearly indicate that the antibacterial pharmacologic activity of Lonicerae flos is markedly different depending on bacteriostatic effect.

The antibacterial effects of Lonicerae flos extracts vary with the different types and methods of preparation. For example,* Lonicera macranthoides* Hand.-Mazz. contains phenolic acids (total chlorogenic acids), glycosides, flavonoids (total flavones), and volatile oil that inhibit* Escherichia coli*,* Staphylococcus aureus*, and* Pseudomonas aeruginosa* to different degrees, with greater inhibition of* Staphylococcus aureus* than* Escherichia coli*. Further, phenolic acids have significantly better antibacterial effects than others [29].

### 3.2. Antiviral Effects

#### 3.2.1. Lonicerae Japonicae Flos

Lonicerae japonicae flos extracts and its active components including chlorogenic acid, flavonoid, caffeoylquinic acid, and iridoid glycoside can inhibit herpes simplex keratitis [30], influenza virus pneumonia [31], influenza A virus [31–33], porcine reproductive and respiratory syndrome virus [34], Newcastle disease virus [35], respiratory syncytial virus [36–38], influenza virus [39], human cytomegalovirus [40], and so on. In addition, the extracts can significantly inhibit and inactivate cytomegalovirus in guinea pigs [41], pseudorabies virus strain Min-A [42], influenza virus variant FM1 [31], Coxsackie *β*_3_ virus [36, 43], enteric cytopathic human orphan 19 virus [43], and so forth. The mechanism of action entails enhancing the binding of drug with ceramidase [44] and increases cellular antiviral potency [45] and organ protection in influenza [46].

#### 3.2.2. Lonicerae Flos

The water extracts of* Lonicera macranthoides* Hand.-Mazz. significantly inhibit the infectivity of 293 cells by defective adenovirus (Ad-lacZ) [45, 46]. The alcohol-soluble component of* Lonicera hypoglauca* Miq. named pheophytin is a hepatitis C virus (HCV) NS3 inhibitor (IC_50_ 0.89 *μ*M), which decreases the expression of HCV viral proteins and ribonucleic acids with dose-dependent effect [47].

The flavone extracts of Lonicerae flos (*Lonicera macranthoides* Hand.-Mazz.) significantly inhibit and inactivate pseudorabies virus (PRV) infection of Vero cells [42]. The active components called chlorogenic acids significantly suppress the Newcastle disease virus (NDV) infection in Vero cells [48] and chlorogenic acids significantly inhibit the proliferation of NDV in Vero cells [49].

### 3.3. Anti-Inflammatory Effects

#### 3.3.1. Lonicerae Japonicae Flos

The water extracts of Lonicerae japonicae flos have significant anti-inflammatory effects on classical inflammatory models such as carrageenan/formaldehyde-induced rat paw swelling [50], mouse ear edema [13, 51], cotton ball granulomatous hyperplasia, mouse cutaneous vascular hyperpermeability [52], and egg white-induced localized acute inflammation [53]. Furthermore, it also exhibits anti-inflammatory effect in rat cervicitis model [54, 55],* Escherichia coli*-infected mouse model [56], excision wound model of infected rat [57], and ovalbumin-induced rat asthma model [58]. The mechanisms include inhibition of inflammatory factor synthesis/release, decreased expression of immune related molecules, and enzyme activities of matrix metalloproteinase.

The water extracts of Lonicerae japonicae flos inhibit the production of histamine and the expression of L-histamine decarboxylase by cultured human keratinocytes [59]. Further, it inhibits the production of nitric oxide and secretion of tumor necrosis factor-alpha (TNF-*α*) by Raw264.7 cells with dose-dependent effects [60] and prevents the trypsin-induced mast cell activation by suppressing the extracellular signal-regulated kinase (ERK) phosphorylation [61]. The polyphenols of* Lonicera japonica* Thunb. downregulate proinflammatory mediators and counter the lipopolysaccharides-induced inflammatory response of Raw264.7 cells by inhibiting NF-*κ*B p65 nuclear translocation and p38 MAPK phosphorylation [62].

According to the Min Jiang research, it is clear that the anti-inflammatory activity of Flos Lonicerae Japonicae (FLJ) has profound material basis. By using UPLC-Q/TOF-MS and dual luciferase reporter gene assay, they revealed the potent NF-*κ*B inhibition influence of the extract from FLJ, which could be classified into 2 types: chlorogenic acid and iridoid glycosides, including swertiamarin. More importantly, as reported in this study, the anti-inflammatory activity decreased during the flowering phases progression. This result suggested that the intensity of anti-inflammatory efficacy of FLJ is dynamically changed in distinct flowering phases, indicating that the effective components temporally affect the clinical application. Taken together, the molecular based quality control (including chlorogenic acid, swertiamarin, and sweroside) and the optimized pharmacological practice are extremely needed and urgent for FLJ [63].

#### 3.3.2. Lonicerae Flos

Water and alcohol extracts of the leaves and flowers of Lonicerae flos have anti-inflammatory effects on dimethylbenzene-induced mouse ear edema, carrageenan-induced rat swelling, and cotton ball granulomatous hyperplasia model [23, 64]. The volatile organic compounds of Lonicerae flos (*Lonicera macranthoides* Hand.-Mazz.) have some inhibitory effects on acetic acid-induced abdominal capillary permeability increase in mice, mouse ear edema, rat pleuritis, and cotton ball granulomatous hyperplasia, significantly lower the concentrations of prostaglandin E_2_ (PGE_2_) and malondialdehyde of the carrageenan-induced paw swelling in norepinephrine animals, and decrease the contents of PGE_2_ and NO in acute pleural effusion of rat [23, 65, 66]. Moreover, the water extracts of Lonicerae flos (*Lonicera macranthoides* Hand.-Mazz.) have significant anti-inflammatory effects by inhibiting the increased capillary permeability in mice, rat paw swelling, and cotton ball granulomatous hyperplasia [25]. The anti-inflammatory effects of both Lonicerae japonicae and Lonicerae flos were listed in Table 2.

### 3.4. Antioxidative Effects

#### 3.4.1. Lonicerae Japonicae Flos

The major antioxidants in Lonicerae japonicae flos include neochlorogenic acid, chlorogenic acid, 4-dicaffeoylquinic acid, caffeic acid, isochlorogenic acid A, isochlorogenic acid B, isochlorogenic acid C, rutin, xylostein, isoquercitrin, luteolin-7-O-glucoside, and luteolin [67, 68].

The alcohol extracts of Lonicerae japonicae flos have antioxidative effects on edible rapeseed oil, peanut oil, ghee, salad oil, mutton tallow [69], linoleic acid, and lard [70]. The crude extracts of chlorogenic acid and total flavones have an inhibitory effect on the antioxidative reaction of oil and prevent the autoxidation of linoleic acid and lard. Further, their redox capacities are 2.0- and 2.8-fold higher than that of synthetic antioxidant butylated hydroxyanisole (BHA) [71, 72]. The antioxidative effects of water and alcohol extracts of Lonicerae japonicae flos on oil may be closely related to the clearance of 2,2-diphenyl-1-picrylhydrazyl (DPPH) and the inhibition of oxygen free radical chain reaction in oil [73, 74].

The ultrasonic-treated extracts and decoction of Lonicerae japonicae flos can both scavenge hydrogen peroxide (H_2_O_2_), hydroxyl radical (^·^OH), and superoxide radical (O^−2^) [75]. The reducing power and the clearance of ^·^OH are positively correlated with the chlorogenic acid content of Lonicerae japonicae flos. Though the alcohol extracts and methanol extracts have better reducing power and higher clearance rates of ^·^OH than water extracts, the water extracts still have a higher clearance rate of DPPH radicals and a stronger chelating ability with Fe^2+^ [76]. In addition, the direct clearance of H_2_O_2_ by water extracts effectively reduces tissue injuries in scalded mice [77]. Analysis of antioxidative effects* in vitro* reveals that the fermented and alcohol extracts of Lonicerae japonicae flos inhibit tyrosinase in mushroom (ED_50_ 4.07 mg/mL and 6.93 mg/mL, resp.). Compared with alcohol extracts, fermented extracts are more effective in promoting the clearance of DPPH (ED_50_ 0.207 mg/mL) and superoxide [78].

Lonicerae japonicae flos significantly upregulates the antioxidant enzyme system of human liver rat basophilic leukemia cell and downregulates NF-*κ*B signal transduction pathway [79, 80]. Moreover, it significantly increases the antioxidant enzyme activities of D-galactose-induced aging model in mice, inhibits lipid peroxidation of liver and kidney tissues, and reduces the oxidative damage in human body [81].

### 3.5. Antipyretic Effects

#### 3.5.1. Lonicerae Japonicae Flos

Lonicerae japonicae flos has antipyretic effects in dry yeast-induced rat fever model [82] and the IL-1*β*-induced fever model in New Zealand rabbits [83], possibly due to the expression of prostaglandin E2 receptor EP3 at the preoptic area of hypothalamus (POAH) neurons [82]. Further, it reduces injuries caused by free radicals and improves human immunity [84].

#### 3.5.2. Lonicerae Flos

The water extracts and alcohol extracts of both* Lonicera macranthoides* Hand.-Mazz. and* Lonicera hypoglauca* Miq. effectively neutralize yeast-induced hyperthermia in rats [64, 85, 86].

### 3.6. Liver Protection

#### 3.6.1. Lonicerae Japonicae Flos

The water extracts of Lonicerae japonicae flos containing 20% chlorogenic acid are protective against alcohol-induced chemical liver injury in mice [87], also it is same liver protection for the water extracts of Lonicerae japonicae flos to the mouse model of acute liver injury induced by intraperitoneal injection of carbon tetrachloride (CCl_4_) [88].

#### 3.6.2. Lonicerae Flos

Two species of Lonicerae flos (*Lonicera fulvotomentosa* Hsu et S. C. Cheng and* Lonicera macranthoides* Hand.-Mazz.) confer different degrees of protection against rat/mouse liver injuries induced by CCl_4_, D-galactosamine (D-Gal), and acetaminophen (AAP) [89]. Saponins of* Lonicera fulvotomentosa* Hsu et S. C. Cheng resolve AAP-induced liver injuries by lowering the cytochrome P450 concentration in liver cells of mouse [90–92]. The total saponins of* Lonicera fulvotomentosa* Hsu et S. C. Cheng significantly alleviate CCl_4_-induced liver injuries, reduce liver injuries of the patient, and effectively lower the incidence of liver necrotizing changes and the total amount of spotty necrosis. Chlorogenic acid has potent choleretic action that not only significantly increases bile secretion volume but also alleviates chromium-induced liver injuries [93, 94].

### 3.7. Immunoregulation

#### 3.7.1. Lonicerae Japonicae Flos

Lonicerae japonicae flos decoction effectively improves human immunity, increases macrophage count, elevates phagocytic ratio and lymphocyte transformation rate [82], and enhances the secretion function of Th1 cells [95]. Lonicerae japonicae flos also promotes the phagocytic function of leucocytes. It decreases T-cell *α*-naphthyl acetate percentage of guinea pig and* in vitro* secretion of neutrophils and remarkably increases the production of IL-2 [96]. The water extracting-alcohol precipitating solution or the flavones of Lonicerae japonicae flos significantly elevate the organ index of immunosuppressed mice [97]. Lonicerae japonicae flos polysaccharides improve mouse splenocyte proliferation [98], markedly enhance immunity, and resolve delayed-type hypersensitivity. Serum hemolysin test shows that Lonicerae japonicae flos polysaccharides enhance humoral immune activities and raise the organ index of immunocompromised animal models, correlated with dosage [99]. Moreover, the water extracts of Lonicerae japonicae flos have significantly regulated immune response in scald-induced immunosuppressive model [100].

The water extracts of Lonicerae japonicae flos effectively substitute the highly toxic immunosuppressants such as cyclosporin A for the induction of immune tolerance. Lonicerae japonicae flos extracts combined with Con A significantly reduce the active degree of T lymphocytes [101] and avoided acute immunological rejection, hence effective in treating graft rejection.

#### 3.7.2. Lonicerae Flos

The water extracting-alcohol precipitating solution of* Lonicera macranthoides* Hand.-Mazz. significantly enhances thymus index, spleen index, carbon clearance, and macrophage phagocytic index in cyclophosphamide-induced immunocompromised mice and improves the proliferation of abdominal macrophages and splenic lymphocytes, with remarkable immunoregulatory effects [91, 102].

### 3.8. Antitumor Effects

#### 3.8.1. Lonicerae Japonicae Flos

Intraperitoneal injection of Lonicerae japonicae flos polysaccharides 30 mg/kg and 90 mg/kg inhibited 23.95% and 30.02% of sarcoma S180, respectively, upregulated the expression level of Bax protein in mouse sarcoma S180, and increased Bax/Bcl-2 ratio and serum TNF-*α* concentration of tumor-bearing mice, indicating an antitumor effect that does not affect the normal growth and immune functions of tumor-bearing mice [103]. The polyphenolic extracts of Lonicerae japonicae flos inhibit proliferation of human hepatoma HepG2 cell line in a dose-dependent manner, decrease the expression of CDK1, CDC25C, cyclin B1, procaspases 3 and 8, and PARP, and promote the phosphorylation of ERK1/2, JNK, and MAPKs and the dephosphorylation of Akt, resulting in G2/M arrest and apoptosis [104].

#### 3.8.2. Lonicerae Flos

The prosapogenin B of* Lonicera macranthoides* Hand.-Mazz. has relatively strong growth inhibition against several types of tumor cells especially HL-60, with a dose of 1.25–5 mg/kg corresponding to a 27.41–54.57% tumor inhibition rate in Lewis tumors. Genechip detection reveals that* Lonicera macranthoides* Hand.-Mazz. induces differential expression of 20 out of 84 tumor-associated genes in HL-60 cells, mainly via cell-cycle arrest and inhibition of cellular invasion and metastasis [105, 106]. The macranthoide B of* Lonicera macranthoides* Hand.-Mazz. inhibit the growth of 6 types of tumor cells, especially HL-60 cells with an IC_50_ of 3.8 *μ*M by activating apoptosis [107]. Studies using high-throughput screening models indicate that the ethyl acetate extracts of* Lonicera macranthoides* Hand.-Mazz. suppress epidermal growth factor receptor (EGFR) kinase, with IC_50_ of 2.027 *μ*g/mL. In addition, the phenolic acids and flavones in the extract may play a major role in inhibiting EGFR kinase [108].

### 3.9. Effects on Glucose and Lipid Metabolism

The water extracts of Lonicerae japonicae flos inhibit the alloxan-induced blood glucose elevation in mice [109]. The results of iodine-starch colorimetry and p-nitrophenyl *α*-D-glucopyranoside (PNPG) assay show that the water extracts dose dependently inhibit activities of *α*-amylase and *α*-glycosidase [110]. Another study indicates that Lonicerae japonicae flos extracts lower the triglyceride levels in serum and liver tissues of hyperlipidemia animal model without significantly affecting serum cholesterol, low-density lipoprotein, high-density lipoprotein, and liver tissue cholesterol [111].

### 3.10. Antiatherosclerotic (As) Effects

The intragastric administration of* Lonicera macranthoides* Hand.-Mazz. decreases the area of atherosclerotic plaque and plaque-to-wall area ratio and alleviates atherosclerotic changes in apolipoprotein E (ApoE) gene knockout mice and reduces lipid droplets and cholesterol concentrations of lipid-loaded THP-1 macrophages [112].

### 3.11. Antiallergic Effects

#### 3.11.1. Lonicerae Japonicae Flos

The caudal vein of egg white lysozyme-sensitized mice with increased blood supply is reduced by 35% alcohol extracts of Lonicerae japonicae flos. This phenomenon indicates that 35% alcohol extracts have antiallergic effects, and the effective components include chlorogenic acid, iridoid, loganin, and sweroside [113].

#### 3.11.2. Lonicerae Flos

The water extracts of* Lonicera fulvotomentosa* Hsu et S. C. Cheng are used to treat the bowel inflammation and ovalbumin- (OVA-) mediated type I hypersensitivity in mice. It also decreases the serum OVA-specific Ig E level of sensitized mice, relieves focal necrosis and abscission of small intestinal villi epithelial cells in mice, reduces the IgA^+^ plasma cell count of small intestinal lamina propria (LP), smIgA^+^ lymphocyte count of Peyer's patches (PP), and mRNA expression of IL-4 of small intestinal LP and PP, increases TGF-*β* mRNA of intestinal mucosa, and decreases the mRNA expression of small intestinal TNF-*α* with dose-dependent effects [114, 115]. Subcutaneous injection of Lonicerae flos total saponins alleviates diarrhea in mice to varying degrees, decreases mast cell aggregation and degranulation, lowers OVA-specific Ig E level, relieves OVA-mediated footpad edema, and resolves small intestinal villi inflammation, indicating that total saponins attenuate mouse Ig E- and immunocyte-mediated hypersensitive response [116, 117]. The volatile organic compounds of* Lonicera macranthoides* Hand.-Mazz. also inhibit heterologous passive cutaneous anaphylaxis of ear and dextran-induced pruritus in mice [118].

### 3.12. Antipregnancy Effects

Intraperitoneal injection of alcohol extracts of Lonicerae japonicae flos has inhibited early pregnancy in mice. Intravenous drip also shows good antipregnancy effects in dogs as early as days 20–22 at a dose-dependent manner [119].

### 3.13. Modulating Gut Microbiota

The water extracts of* Lonicera fulvotomentosa* Hsu et S. C. Cheng significantly improve intestinal folate deficiency and reduce Gram-negative bacterial resistance and intestinal flora imbalance in rat obstructive jaundice model [120]. The water extracts also promote the growth of bifidobacteria and lactobacilli* in vitro*, but they inhibit the growth at high concentration [121].

### 3.14. Antiultraviolet Radiation

Administration of water extracting-alcohol precipitating solution of Lonicerae japonicae flos reduces the breakage of wavy elastic fibers in the skin and the coiling degree of mouse model with ultraviolet radiation-aged skin injuries, possibly due to the antioxidant effects [122].

### 3.15. Antiendotoxin Effects

The water extracts 10 mg/mL of Lonicerae japonicae flos and chlorogenic acids 1 mg/mL destroy the ultrastructure of endotoxin [123].

### 3.16. Spasmolytic Effects

The decoction of Lonicerae japonicae flos inhibits the motility of isolated small intestine from rabbit and significantly reduces rabbit small intestinal smooth muscle contraction, electrical activity range (IC_50_, 6.30 mg/mL), frequency, and area under curve in dose-dependent manner. Propranolol, L-NAME, and glibenclamide partly block the inhibitory effects of Lonicerae japonicae flos on rabbit small intestinal smooth muscle contraction [124]. The water extracting-alcohol precipitating solution of Lonicerae japonicae flos also inhibits the motility of rabbit isolated small intestine [125]. The decoction of Lonicerae japonicae flos significantly inhibits acetylcholine-induced intra- and extracellular calcium-mediated smooth muscle contraction [124].

### 3.17. Antiplatelet Effect

The water extracts of Lonicerae japonicae flos inhibited ADP-induced platelet aggregation with IC_50_ of 0.028 g/L. The IC_50_ of the organic acids in the water extracts are as follows: isomers of chlorogenic acid (4-caffeoylquinic acid, 0.0286 g/L; 5-caffeoylquinic acid, 1.707 g/L), caffeic acid (2.411 g/L), and isochlorogenic acids (3,4-dicaffeoylquinic acid, 0.026 g/L; 3,5-dicaffeoylquinic acid, 0.328 g/L; and 4,5-dicaffeoylquinic acid, 0.539 g/L), indicating relatively strong antiplatelet aggregation effects [126]. Extracts from Lonicerae japonicae flos, methyl caffeate (a polyphenol), methyl chlorogenic acid, digicitrin, and so on significantly inhibited superoxide-induced platelet activation and cellular injuries, ADP-induced platelet aggregation, and calcium ionophore A23187-triggered thromboxane synthesis in platelet microparticles [127]. Methyl caffeate, 3,4-di-O-caffeoylquinic acid, and methyl 3,4-di-O-caffeoylquinate have relatively strong inhibitory effect on platelet aggregation [128].

### 3.18. Neuroprotective Effect

The water extracts of Lonicerae japonicae flos have potential antiparkinsonian activities and reduce 6-hydroxydopamine-induced SH-SY5Y cytotoxicity. The protective mechanism is closely related to the inhibition of cellular apoptosis and oxidative stress and the activation of MAPKs, PI3K/Akt, and NF-*κ*B pathways [129]. Further, the water extracts neutralize the H_2_O_2_-induced SH-SY5Y neuroblastoma cytotoxicity, apoptosis, and ROS production to protect the nerves, possibly via inhibition of Akt, JNK, p38 MAPK, and ERK1/2 phosphorylation [130]. The fluorescence spectrum analysis using thioflavin-T fluorometric assay and atomic force microscopy reveals that the dextran derived from Lonicerae japonicae flos inhibits A*β*_42_ deposition with dose-dependent effects and reduces the neurotoxicity of A*β*_42_ for SH-SY5Y cells, suggesting potential therapeutic value for Alzheimer's disease [131].

### 3.19. Toxicity and Adverse Effects

Intragastric administration of aqueous extracts of Lonicerae japonicae flos is not significantly toxic and does not affect the respiration, blood pressure, or urine volume in experimental animals. The LD_50_ of subcutaneous injection of Lonicerae japonicae flos concrete is 53 g/kg for mice [132]. The LD_50_ of oral administration of Lonicerae japonicae flos is larger than 15 g/kg and thus innocuous. The micronucleus test on bone marrow polychromatic erythrocytes of mice and Ames* Salmonella*/microsome mutagenicity assay do not reveal any mutagenicity of Lonicerae japonicae flos. Sperm shape abnormality tests in mice reveal no genotoxicity of Lonicerae japonicae flos for germ cells of male animals. The anti-early pregnancy assay of SD rat (oral administration) indicates no adverse effects of Lonicerae japonicae flos on the reproductive function of female animals during pregnancy [133].

After intragastric administration of* Lonicera macranthoides* Hand.-Mazz. decoction, the mice show significantly decreased spontaneous activities and some degree of sleepiness and prone position, which were restored in 24 hours. Most death occurred in 24 hours, before any convulsions or seizure. However, subsequent anatomical observation indicates no lesions in major organs and the LD_50_ was 73.95 (69.80–78.34) g/kg [134]. The maximum dosage of* Lonicera macranthoides* Hand.-Mazz. buds for mice is 15 g/kg, and subacute toxicity test shows no dose-related differences in weight, hematology, blood biochemistry, or organ index with normal control group [135].

## 4. Comparative Pharmacology and Toxicology of Lonicerae Japonicae Flos and Lonicerae Flos

According to pharmacopoeia, Lonicerae japonicae flos and Lonicerae flos have similar efficacy, but researchers still compare the pharmacology and toxicity experimentally since the drugs have different origin and geographic distribution.

### 4.1. Comparison of Antibacterial and Bacteriostatic Effects

*Lonicera macranthoides* Hand.-Mazz. and Lonicerae japonicae flos have similar antibacterial spectra that significantly inhibit and kill* Staphylococcus aureus*,* Escherichia coli*,* Salmonella* Typhi,* Shigella dysenteriae*,* Proteus vulgaris*,* Streptococcus* group B,* Sarcina*, and* Bacillus subtilis*, but their effects are still different. The former has more significantly inhibitory effects on* Streptococcus* group B [27],* Escherichia coli*,* Staphylococcus aureus*, and* Pneumococcus* [136] than Lonicerae japonicae flos. Only* Lonicera macranthoides* Hand.-Mazz. has germicidal effects on* Shigella dysenteriae* [137]. Compared with Lonicerae japonicae flos, it has better bactericidal effects on* Shigella dysenteriae* [44, 137],* Sarcina*, and* Bacillus subtilis* [44] and poorer effects on* Salmonella* Typhi [44] and* Escherichia coli* [138] while having similar effects on* Pseudomonas aeruginosa* [138].* Lonicera confusa* DC. exhibits inhibitory effect against* Staphylococcus aureus* and* Salmonella* Typhi, equivalent to that of Lonicerae japonicae flos, but better than that of* Lonicera hypoglauca* Miq. Moreover, it has more significant inhibitory effects on* Streptococcus haemolyticus* than Lonicerae japonicae flos and* Lonicera hypoglauca* Miq. [139].* Lonicera macranthoides* Hand.-Mazz. decoction 40 g/kg is protective in* Staphylococcus aureus*-infected mice, with a significantly longer survival time than Lonicerae japonicae flos [140]. In conclusion, all species of Lonicerae flos are more antibacterial and bacteriostatic.

### 4.2. Comparison of Antiviral Effects

The flavone extracts of* Lonicera macranthoides* Hand.-Mazz. and Lonicerae japonicae flos are significantly inhibitory against pseudorabies virus- (PRV-) infected Vero cells, with* Lonicera macranthoides* Hand.-Mazz. showing a stronger effect than Lonicerae japonicae flos, but not significantly different in blocking effects [42]. The chlorogenic acid extracts of* Lonicera macranthoides* Hand.-Mazz. and Lonicerae japonicae flos inhibit Newcastle disease virus that affects Vero cells without any notable differences in efficacy [48].* Lonicera macranthoides* Hand.-Mazz. and the alcohol extracts of Lonicerae japonicae flos remarkably enhance the antiadenoviral ability of cells* in vitro*, not significantly different [45].

### 4.3. Comparison of Antioxidant Effects

Orthophenanthroline-Fe^2+^ colorimetry is used to compare the scavenging capacity of ^·^OH* in vitro* between the water extracts of* Lonicera macranthoides* Hand.-Mazz. and Lonicerae japonicae flos, which reveals equally strong scavenging capacity of ^·^OH (IC_50_ of scavenging rate,* Lonicera macranthoides* Hand.-Mazz.* versus* Lonicerae japonicae flos, 6.01 mg/mL* versus* 10.22 mg/mL) [136]. Biochemiluminescence indicates that the IC_50_ of luminescence inhibition rates of O^−2^ and ^·^OH were 10.26 mg/mL and 3.26 mg/mL for the water extracts of* Lonicera macranthoides* Hand.-Mazz. and 16.48 mg/mL and 10.79 mg/mL for Lonicerae japonicae flos [141]. The total flavones of* Lonicera hypoglauca* Miq. inhibit lipid oxidation (according to peroxide value test). The detection results of H_2_O_2_-CTMAB-luminol fluorescence system show an IC_50_ of fluorescence inhibition rate of 2.21 × 10^−2 ^mg/mL for the total flavones of* Lonicera hypoglauca* Miq. and 1.54 × 10^−2^ mg/mL for Lonicerae japonicae flos, indicating different H_2_O_2_ scavenging capacities [142].

### 4.4. Comparison of Anti-Inflammatory Effects

*Lonicera macranthoides* Hand.-Mazz. and Lonicerae japonicae flos used at 1 and 10 g/kg significantly inhibit the abdominal capillary permeability in mice and suppress carrageenan-induced paw swelling, with no significant differences. Lonicerae japonicae flos 10 g/kg inhibits dimethylbenzene-induced ear edema in mice but* Lonicera macranthoides* Hand.-Mazz. only shows inhibitory trends and the comparison between them reveals significant difference [44, 138].

### 4.5. Comparison of Antipyretic Effects

*Lonicera macranthoides* Hand.-Mazz. and Lonicerae japonicae flos 20 g/kg equally inhibit the fever induced by subcutaneous injection of* Saccharomyces cerevisiae* in mice, although the latter is effective for a longer time [140].* Lonicera macranthoides* Hand.-Mazz., 10 g/kg, shows inhibitory trend against yeast powder-induced fever in mice and the antipyretic effect is slightly weaker than Lonicerae japonicae flos at the same dose [138, 140].

### 4.6. Comparison of Immunoregulatory Effects

*Lonicera macranthoides* Hand.-Mazz. and the water extracting-alcohol precipitating concentrated concretes of Lonicerae japonicae flos (main components: total flavones and chlorogenic acid) 1 g/kg and 10 g/kg significantly increase spleen index, thymus index, carbon clearance index, and phagocytic index in mice, but not significantly differently [44, 138]. Lonicerae japonicae flos 10 g/kg significantly elevates the white cell count in normal and immunocompromised mice unlike* Lonicera macranthoides* Hand.-Mazz. [138].

The water extracts of* Lonicera fulvotomentosa* Hsu et S. C. Cheng alleviate small intestinal villi inflammation in sensitized mice, reduce mast cell aggregation and cell degranulation, increase the whole-mast cell ratio in LP, decrease intestinal histamine release of sensitized mice, lower the levels of IL-4 and OVA-specific Ig E levels in sensitized mice, and resolved OVA-mediated delayed-type hypersensitivity of footpad in mice [143].

### 4.7. Comparison of Hemostatic Effects

Both Lonicerae japonicae flos and Lonicerae flos (*Lonicera confusa* DC. and* Lonicera hypoglauca* Miq.) shorten bleeding time in mice. Lonicerae japonicae flos has similar hemostatic effect equivalent to* Lonicera confusa* DC. Lonicerae japonicae flos and* Lonicera confusa* DC. are significantly more hemostatic than* Lonicera hypoglauca* Miq. [139].

### 4.8. Comparison of Toxicity and Adverse Effects

According to a comparative study, the LD_50_ values of* Lonicera macranthoides* Hand.-Mazz. and Lonicerae japonicae flos are 84.14 g/kg and 72.95 g/kg, respectively, with no significant difference [27].

The water extracts of sun-cured* Lonicera macranthoides* Hand.-Mazz. are hemolytic. However, the water extracts of steaming sun-cured* Lonicera macranthoides* Hand.-Mazz. and Lonicerae japonicae flos show hemolytic reaction only after 3 hours [144]. Total saponins of* Lonicera fulvotomentosa* Hsu et S. C. Cheng also result in mild hemolysis [139].

In active systemic anaphylaxis test using crude water extracting-alcohol precipitating solution of* Lonicera macranthoides* Hand.-Mazz., four out of six sensitized guinea pigs show dyspnea, gait instability, and Cheyne-Stokes respiration and die in 40 s to 5 min after excitation by intravenous injection of chlorogenic acid 15 mg/kg. Two out of six guinea pigs show agitation, rapid respiration, and gait instability in 30 s. In 10 to 15 min, these symptoms disappear and the mice recover. The allergic reaction intensity is graded as strongly positive. After intravenous injection of crude extracts 15 mg/kg, the nonsensitized guinea pigs manifest agitation, gait instability, rapid respiration, spasm, urination, and defecation in 10 s to 2.5 min and recover in 20–30 min. The strength of anaphylactic reaction is strongly positive [145].

Some researchers have compared the hypersensitive and anaphylactic reactions of Lonicerae japonicae flos and Lonicerae flos. The intraperitoneal injection of the water extracts and water extracting-alcohol precipitating solution of Lonicerae japonicae flos or* Lonicera macranthoides* Hand.-Mazz. on alternate days for 3 times may lead to hypersensitive reaction in guinea pigs, with possible death. A further study reveals that the degranulation rate (based on *β*-hexosaminidase release assay) of rat basophilic leukemia cells (RBL-2H) induced by the water extracting-alcohol precipitating solution of* Lonicera macranthoides* Hand.-Mazz. is significantly higher than that of Lonicerae japonicae flos (11.33% ± 0.78* versus *8.52% ± 0.44), but with similar activation and proliferation inhibition on peripheral blood mononuclear cells [146].

Therefore, compared with Lonicerae japonicae flos, Lonicerae flos may be potentially dangerous.

## 5. Summary

*Chinese Pharmacopeia* (1963 Edition) records* Lonicera japonica* Thunb. of Caprifoliaceae family as the only plant source of medicinal Lonicerae japonicae flos, after which a total of 5 editions of pharmacopeias included the origin of Lonicerae japonicae flos and 4 plants of the same genus under the legal species of Lonicerae japonicae flos. In this systematic review, we confirm that the medicinal value of Lonicerae japonicae flos is limited to* Lonicera japonica* Thunb. of Caprifoliaceae family in traditional TCM books without any historical evidence supporting the medicinal use of Lonicerae flos-related species, thus providing a scientific basis for the independent listing of Lonicerae japonicae flos and Lonicerae flos since* Chinese Pharmacopeia* (2005 Edition).

The functions and indications of Lonicerae japonicae flos and Lonicerae flos are similar in* Chinese Pharmacopeia* 2005 and 2010 Editions. Our results confirm similar pharmacological activities of Lonicerae japonicae flos and Lonicerae flos, but the former is more widely studied pharmacologically. Lonicerae japonicae flos has glucose-lowering, anti-early pregnancy, antiultraviolet radiation, antiendotoxin, antiulcer, antiplatelet aggregation, antifertility, and neuroprotective activities that are not reported in Lonicerae flos.* Lonicera macranthoides* Hand.-Mazz. and Lonicerae japonicae flos have similar antibacterial, antiviral, anti-inflammatory, antioxidative, antifebrile, hepatoprotective, immunoregulatory, and antitumor activities. However, the pharmacological effects involving balancing intestinal flora and antiatherosclerotic effects have not been reported in Lonicerae japonicae flos. Antioxidation is the common pharmacological activity of* Lonicera hypoglauca* Miq.,* Lonicera confusa* DC., and* Lonicera fulvotomentosa* Hsu et S. C. Cheng. In addition,* Lonicera hypoglauca* Miq. also has antibacterial, anti-inflammatory, and antipyretic effects;* Lonicera confusa* DC. has antibacterial and hemostatic effects;* Lonicera fulvotomentosa* Hsu et S. C. Cheng has hepatoprotective and antiallergic effects and saponins have mild hemolytic effect.

Further analysis reveals twenty plus studies comparing the pharmacological activities between Lonicerae japonicae flos and Lonicerae flos. Lonicerae flos has certain advantages in terms of antibacterial and other effects. Some studies report better antibacterial and bacteriostatic activities with* Lonicera macranthoides* Hand.-Mazz. and* Lonicera confusa* DC. than Lonicerae japonicae flos and more antioxidant activities of* Lonicera macranthoides* Hand.-Mazz. than Lonicerae japonicae flos.* Lonicera macranthoides* Hand.-Mazz. and Lonicerae japonicae flos do not differ significantly in antiviral effects.* Lonicera confusa* DC. and Lonicerae japonicae flos do not differ significantly in hemostatic effect but both are better than* Lonicera hypoglauca* Miq. Lonicerae japonicae flos is slightly better than* Lonicera macranthoides* Hand.-Mazz. in anti-inflammatory, antipyretic, and immunoregulatory effects, but no significant difference in toxicity. Some studies have reported adverse events such as hypersensitive/anaphylactic and hemolytic reaction in* Lonicera macranthoides* Hand.-Mazz. and* Lonicera fulvotomentosa* Hsu et S. C. Cheng. Therefore, meticulous screening and identification of the different species are essential to avoid the risk of adverse and toxic effects during the production and clinical use.

In conclusion, modern pharmacological effects of Lonicerae japonicae flos and Lonicerae flos are similar, although a few significant differences should not be neglected. Since a systematic and standard comparative study has not been developed so far, it is difficult to scientifically evaluate their advantages/disadvantages and differences/similarities. We suggest a comprehensive systematic review and a parallel, crossover study to delineate the mechanisms underlying the comparative pharmacological activities of Lonicerae japonicae flos and Lonicerae flos and different species of Lonicerae flos. A comparative analysis of the clinical efficacy and safety of pharmacologically active ingredients/products in Lonicerae japonicae flos and Lonicerae flos is essential to fully and accurately evaluate their effects and toxic side effects. References for the revision of relevant pharmacopoeial records should be provided along with supporting clinical efficacy and safety data.

## Figures and Tables

**Table 1:** Antibacterial effects of Lonicerae japonicae flos and Lonicerae flos.

Variety	Part/compound	Antibacterial spectrum		Detection methods	References
Lonicerae japonicae flos	Water extract	Gram-positive bacteria	*Streptococcus mutans*, *Actinomyces viscosus*, *Staphylococcus aureus*, *Bacillus subtilis*, pathogenic *Streptococcosis* of recurrent aphthous ulcer	Dilution-plate method (for minimum inhibitory concentration [MIC]); filter paper method; agar diffusion method	[147–149]
		Gram-negative bacteria	Black-pigmented bacteria, *Pseudomonas aeruginosa* P29, *Pseudomonas aeruginosa*, *Escherichia coli *	Dilution-plate method (for MIC); replica plating method, elimination experiment; *in vivo *test; tube dilution method (for MIC); filter paper method	[19, 147, 148, 150]
		Fungi	*Penicillium*, *Aspergillus flavus*, *Aspergillus niger*, *Epidermophyton floccosum*, *Trichophyton violaceum*, *Trichophyton schoenleinii*, *Ashland's Grubyella variant Mongolia*, *Concentric endodermophyton*, *Trichophyton interdigitalis*, *ToeTrichophyton*, dog* Microsporum*, *Ferruginous microsporum*, *Nocardia asteroides *	Filter paper method; fungal inhibition test	[18, 148]
		Drug-resistant bacteria	MRSA, MRSH, MRSE, HLAR	Dilution-plate method (for MIC)	[22]
	Water extracting-alcohol precipitating solution	Gram-positive bacteria	*Staphylococcus aureus *	Microdilution checkerboard method	[151]
		Gram-negative bacteria	*Salmonella*, *Escherichia coli *	Microdilution checkerboard method	[151]
	Water extract and alcohol extract	Gram-positive bacteria	*Streptococcus suis* type 2	Microtubule-plate method	[152]
	Water-soluble polysaccharide	Gram-positive bacteria	*Bacillus subtilis*, *Staphylococcus aureus *	Circular paper method and MIC detection	[16]
		Gram-negative bacteria	*Escherichia coli*, *Proteus vulgaris *	Circular paper method and MIC detection	[16]
		Fungi	*Saccharomyces cerevisiae*, *Penicillium citrinum*, *Aspergillus niger *	Circular paper method and MIC detection	[16]
	Supercritical carbon dioxide extract	Gram-positive bacteria	*Staphylococcus aureus*, *Bacillus subtilis*, *Staphylococcus albus *	Agar diffusion method and tube dilution method (for MIC)	[14]
		Gram-negative bacteria	*Escherichia coli*, *Pseudomonas aeruginosa*, *Salmonella *Typhimurium		
	Total isochlorogenic acid, total chlorogenic acid, total flavone, total iridoid glycoside	Gram-negative bacteria	*Escherichia coli *	Microcalorimetric method	[153]
	Chlorogenic acid	Gram-positive bacteria Gram-negative bacteria	*Sarcina lutea*, *Bacillus subtilis* *Escherichia coli *	Dilution method (for MIC)	[154]

Lonicerae flos	*Lonicera hypoglauca* Miq. alcohol extract 70%	Gram-positive bacteria	*Staphylococcus aureus *	Agar dilution method (for MIC)	[23]
		Gram-negative bacteria	*Escherichia coli*, *Pseudomonas aeruginosa *		
	Water extracting-alcohol precipitating solution of *Lonicera macranthoides *Hand.-Mazz.	Gram-positive bacteria	*Staphylococcus aureus*, *Sarcina*, *Bacillus subtilis *	Disk agar diffusion method	[155]
		Gram-negative bacteria	*Escherichia coli*, *Pseudomonas aeruginosa*, *Salmonella *Typhi		
	Water extract of *Lonicera macranthoides* Hand.-Mazz. plumule	Gram-positive bacteria	*Staphylococcus aureus*, *Sarcina micrococcus*, *Bacillus subtilis *	Slip method	[25]
		Gram-negative bacteria	*Escherichia coli*, *Pseudomonas aeruginosa*, *Salmonella *Typhi		
	*Lonicera macranthoides* Hand.-Mazz. components: phenolic acid (total chlorogenic acid), glycoside, flavonoid (total flavone), and volatile oil	Gram-positive bacteria	*Staphylococcus aureus *	Slip method	[29]
		Gram-negative bacteria	*Escherichia coli*, *Pseudomonas aeruginosa *		
	Water extract of *Lonicera hypoglauca* Miq.	Gram-positive bacteria	*Staphylococcus aureus*, *Streptococcus haemolyticus*, *Streptococcus pneumoniae *	Punch method	[28]
		Gram-negative bacteria	*Escherichia coli*, *Shigella flexneri*, *Salmonella *Typhimurium		

**Table 2:** Anti-inflammatory effects of Lonicerae japonicae flos and Lonicerae flos.

Species	Part/compound	Detection methods	References
Lonicerae japonicae flos	Water extract	Carrageenan-induced paw swelling model, dimethylbenzene/formalin-induced inflammatory model, cotton ball granulomatous hyperplasia model, cervicitis model, *Escherichia coli* infection model, Raw264.7 cell activation model, trypsin-induced mast cell activation model, human keratinocyte three-dimensional culture inflammatory activation model	[50, 52, 54, 56, 59–61]
	Alcohol extract 30%	Croton oil-induced inflammatory model	[55]
	Alcohol extract 57%	Egg white-induced paw swelling model, dimethylbenzene-induced inflammatory model	[51, 53]
	Alcohol extract 70%	LPS-induced Raw264.7 cell activation model	[62]
	Alcohol extract 95%	Resection wound model, H1N1-infected human bronchial epithelial cell line A549 model	[57, 156]
	Alcohol extract	Ovalbumin-induced asthma model	[58]
	Supercritical carbon dioxide extract	Dimethylbenzene-induced inflammatory model	[51]

Lonicerae flos	Water extract of *Lonicera macranthoides* Hand.-Mazz.	Formalin pain model, acetic acid-writhing model, xylene-induced inflammation model, carrageenan-induced paw swelling model, yeast thermal model	[85]
	Water extract of *Lonicera macranthoides* Hand.-Mazz. flower	Acetic acid-induced capillary permeability increase model, paw swelling model, cotton ball granulomatous hyperplasia model	[102]
	Supercritical carbon dioxide extract of *Lonicera macranthoides* Hand.-Mazz.	Acetic acid-induced abdominal capillary permeability increase model, carrageenan-induced paw swelling model, pleuritis model, cotton ball granulomatous hyperplasia model	[65, 66]
	Water extract 50% and alcohol extract 95% of *Lonicera hypoglauca* Miq.	Dimethylbenzene-induced inflammation model, carrageenan-induced paw swelling model, cotton ball granulomatous hyperplasia model	[64]
